# Supplementary material for: Personalized lead exposure information and preventive behaviors in Ivory Coast: Insights from a pilot study
Source: PLoS One. 2025 Nov 14;20(11):e0336949. doi: 10.1371/journal.pone.0336949 (PMC12617878; doi:10.1371/journal.pone.0336949)
Supplement: S1 Fig — (PDF) [file pone.0336949.s001.pdf]

# Leaflet on Pb risks, sources of Pb exposure and preventive measures.

Figure 1: Generic information on lead

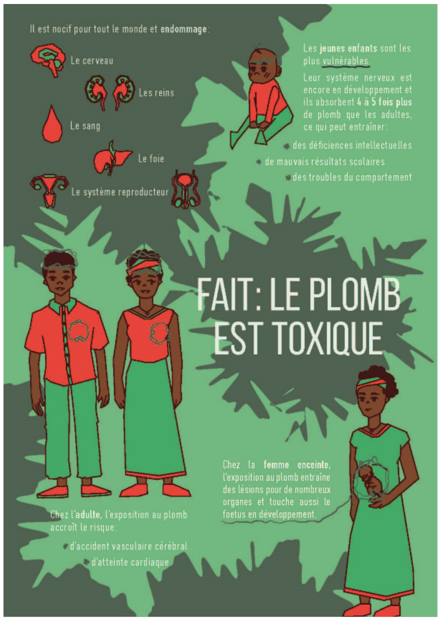


(a) Health impacts of lead exposure

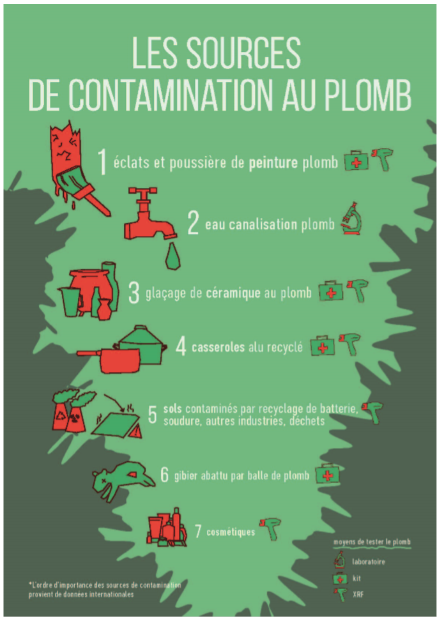


(b) Sources of lead exposure

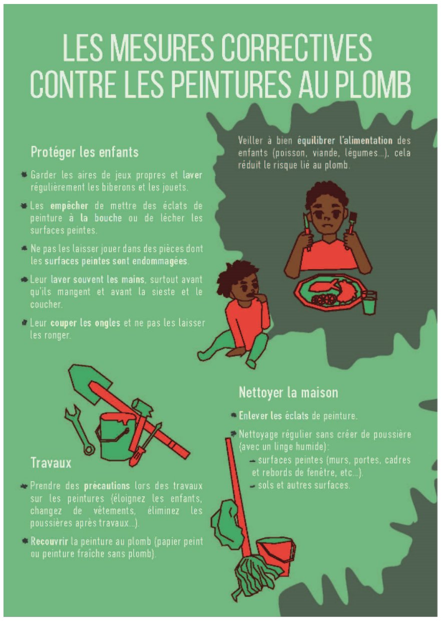


(c) Preventive measures against exposure from lead in paint

Note : The figures were designed by the project team for this research
