# Supplementary material for: Personalized lead exposure information and preventive behaviors in Ivory Coast: Insights from a pilot study
Source: PLoS One. 2025 Nov 14;20(11):e0336949. doi: 10.1371/journal.pone.0336949 (PMC12617878; doi:10.1371/journal.pone.0336949)
Supplement: S1 Table — (PDF) [file pone.0336949.s002.pdf]

Table 1: Attrition Analysis

	Endline resp.		Attriters (Wave 1–3)		Diff. Attriters & Endline	
	Mean	Std err.	Mean	Std err.	Mean	Std err.
	(1)	(2)	(3)	(4)	(5)	(6)
**Women characteristics**						
First trimester pregnancy	0.57	0.50	0.47	0.50	-0.10	0.08
Has a partner	0.87	0.34	0.74	0.44	-0.12**	0.06
Age	28.37	6.13	26.81	5.79	-1.56	1.01
No education	0.30	0.46	0.28	0.45	-0.02	0.08
Primary education	0.28	0.45	0.13	0.34	-0.15**	0.07
Secondary education	0.29	0.45	0.32	0.47	0.03	0.08
Tertiary education	0.13	0.34	0.28	0.45	0.15**	0.06
Salaried	0.11	0.32	0.19	0.40	0.08	0.06
Self-Employed	0.46	0.50	0.36	0.49	-0.10	0.08
No work	0.42	0.50	0.45	0.50	0.02	0.08
**House characteristics**						
Nb. rooms	2.68	1.28	2.72	1.66	0.04	0.23
**Household characteristics**						
Household size	3.86	1.57	3.38	1.42	-0.47	0.26
Nb. children < 5 yrs	0.58	0.64	0.40	0.61	-0.17	0.11
Wealth score	-0.04	1.78	0.11	1.71	0.15	0.29
House owner	0.07	0.26	0.11	0.31	0.03	0.05
**Pb knowledge**						
Pb exposure is dangerous	0.30	0.46	0.34	0.48	0.04	0.08
Paint is a source of Pb exposure	0.03	0.16	0.02	0.15	-0.00	0.03
Exposed to Pb	0.17	0.38	0.15	0.36	-0.02	0.06
Demonstration test: lead in paint	0.59	0.49	0.57	0.50	-0.02	0.08
**Behavior**						
Clean house at least twice p.w.	0.90	0.31	0.89	0.31	-0.00	0.05
Use mop at least twice p.w.	0.84	0.37	0.89	0.31	0.06	0.06
Washed toys in last two w.	0.26	0.44	0.38	0.51	0.12	0.14
Wash kids' hands bf. eat	0.80	0.40	0.92	0.28	0.12	0.12
Wash kids' hands bf. sleep	0.10	0.30	0.23	0.44	0.13	0.10
Wash kids' hands af. outside	0.30	0.46	0.31	0.48	0.01	0.14
Nb. observations	153		47			

Note: This table reports the means and standard errors of baseline characteristics for respondents present at endline (Columns 1–2) and for attriters between Wave 1 and Wave 3 (Columns 3–4). Columns 5–6 report the mean differences between the two groups, along with the corresponding standard errors. * $p < 0.1$, ** $p < 0.05$, *** $p < 0.01$. We do not report the number of painted surfaces, which is only measured for the sample of respondents present at endline.
