# Supplementary material for: Personalized lead exposure information and preventive behaviors in Ivory Coast: Insights from a pilot study
Source: PLoS One. 2025 Nov 14;20(11):e0336949. doi: 10.1371/journal.pone.0336949 (PMC12617878; doi:10.1371/journal.pone.0336949)
Supplement: S2 Table — (PDF) [file pone.0336949.s003.pdf]

Table 1: Perceived and Known Lead Exposure

	Do you think that you are exposed to lead in your daily life?			I know there is lead		
	Outcome: Yes			Outcome: Yes		
	(1)	(2)	(3)	(4)	(5)	(6)
Asset based wealth score	0.00	0.01	0.01	-0.02	-0.02	-0.02
	(0.89)	(0.84)	(0.73)	(0.45)	(0.44)	(0.51)
	[0.02]	[0.02]	[0.02]	[0.03]	[0.03]	[0.03]
	[-0.04,0.05]	[-0.04,0.05]	[-0.04,0.06]	[-0.08,0.04]	[-0.08,0.04]	[-0.07,0.04]
No education (Head)	-0.14	-0.15	-0.14	-0.00	-0.02	-0.01
	(0.20)	(0.17)	(0.17)	(0.99)	(0.87)	(0.91)
	[0.11]	[0.11]	[0.10]	[0.13]	[0.13]	[0.12]
	[-0.35,0.07]	[-0.36,0.07]	[-0.35,0.06]	[-0.25,0.25]	[-0.27,0.23]	[-0.26,0.23]
Primary educ (Head)	-0.01	-0.01	-0.03	-0.09	-0.10	-0.12
	(0.93)	(0.91)	(0.73)	(0.44)	(0.41)	(0.29)
	[0.10]	[0.10]	[0.10]	[0.12]	[0.12]	[0.12]
	[-0.21,0.19]	[-0.22,0.19]	[-0.23,0.16]	[-0.33,0.15]	[-0.34,0.14]	[-0.35,0.11]
Sec. educ (Head)	0.02	0.00	0.01	-0.04	-0.07	-0.06
	(0.83)	(0.98)	(0.89)	(0.68)	(0.50)	(0.56)
	[0.08]	[0.09]	[0.08]	[0.10]	[0.10]	[0.10]
	[-0.15,0.19]	[-0.17,0.17]	[-0.15,0.17]	[-0.24,0.16]	[-0.27,0.13]	[-0.25,0.14]
No educ (Woman)	0.04	0.06	-0.01	0.03	0.07	-0.00
	(0.72)	(0.60)	(0.94)	(0.80)	(0.57)	(0.99)
	[0.11]	[0.11]	[0.11]	[0.13]	[0.13]	[0.13]
	[-0.18,0.26]	[-0.16,0.28]	[-0.22,0.21]	[-0.22,0.29]	[-0.18,0.33]	[-0.25,0.25]

Primary educ (Woman)	0.13	0.15	0.07	0.08	0.12	0.03
	(0.20)	(0.15)	(0.51)	(0.50)	(0.33)	(0.83)
	[0.11]	[0.11]	[0.11]	[0.13]	[0.13]	[0.12]
	[-0.07,0.34]	[-0.06,0.37]	[-0.14,0.28]	[-0.16,0.33]	[-0.13,0.37]	[-0.22,0.27]
Sec. educ (Woman)	0.18	0.19	0.12	0.20	0.23	0.14
	(0.07)	(0.05)	(0.23)	(0.09)	(0.05)	(0.21)
	[0.10]	[0.10]	[0.10]	[0.12]	[0.12]	[0.11]
	[-0.02,0.37]	[-0.00,0.39]	[-0.08,0.31]	[-0.03,0.43]	[0.00,0.46]	[-0.08,0.37]
35-30 years old	0.00	0.02	0.01	-0.01	0.02	0.01
	(0.98)	(0.80)	(0.95)	(0.92)	(0.79)	(0.93)
	[0.08]	[0.08]	[0.08]	[0.09]	[0.09]	[0.09]
	[-0.15,0.16]	[-0.14,0.18]	[-0.15,0.16]	[-0.19,0.17]	[-0.16,0.21]	[-0.17,0.19]
30-35 years old	0.03	0.05	0.04	-0.12	-0.09	-0.10
	(0.78)	(0.63)	(0.69)	(0.27)	(0.43)	(0.35)
	[0.09]	[0.09]	[0.09]	[0.11]	[0.11]	[0.11]
	[-0.16,0.21]	[-0.14,0.23]	[-0.14,0.21]	[-0.34,0.09]	[-0.30,0.13]	[-0.31,0.11]
35-40 years old	0.08	0.09	0.07	0.10	0.12	0.10
	(0.47)	(0.40)	(0.49)	(0.42)	(0.35)	(0.43)
	[0.11]	[0.11]	[0.11]	[0.13]	[0.13]	[0.12]
	[-0.14,0.29]	[-0.12,0.31]	[-0.14,0.28]	[-0.15,0.36]	[-0.13,0.37]	[-0.15,0.34]
40-45 years old	-0.05	-0.04	-0.01	-0.01	0.01	0.05
	(0.72)	(0.77)	(0.95)	(0.97)	(0.94)	(0.76)
	[0.15]	[0.15]	[0.14]	[0.18]	[0.18]	[0.17]
	[-0.35,0.24]	[-0.34,0.25]	[-0.29,0.27]	[-0.36,0.34]	[-0.33,0.36]	[-0.28,0.39]
Nb child. $\leq$ 5 yrs old	0.06	0.04	0.04	0.06	0.04	0.04
	(0.30)	(0.50)	(0.45)	(0.36)	(0.57)	(0.53)

	[0.06]	[0.06]	[0.06]	[0.07]	[0.07]	[0.07]
	[-0.05,0.17]	[-0.08,0.16]	[-0.07,0.16]	[-0.07,0.20]	[-0.10,0.18]	[-0.09,0.18]
Two months pregnant	0.23	0.24	0.30	0.10	0.12	0.19
	(0.07)	(0.07)	(0.02)	(0.52)	(0.44)	(0.20)
	[0.13]	[0.13]	[0.12]	[0.15]	[0.15]	[0.15]
	[-0.02,0.48]	[-0.02,0.49]	[0.05,0.54]	[-0.20,0.40]	[-0.18,0.41]	[-0.10,0.48]
Three months pregnant	0.09	0.10	0.16	0.03	0.05	0.12
	(0.42)	(0.37)	(0.14)	(0.82)	(0.71)	(0.36)
	[0.11]	[0.11]	[0.11]	[0.13]	[0.13]	[0.13]
	[-0.13,0.31]	[-0.12,0.32]	[-0.05,0.37]	[-0.23,0.29]	[-0.21,0.31]	[-0.13,0.37]
Four months pregnant	0.12	0.14	0.16	0.25	0.29	0.31
	(0.30)	(0.24)	(0.16)	(0.08)	(0.04)	(0.02)
	[0.12]	[0.12]	[0.12]	[0.14]	[0.14]	[0.14]
	[-0.11,0.36]	[-0.10,0.38]	[-0.06,0.39]	[-0.03,0.53]	[0.01,0.57]	[0.04,0.58]
Five months pregnant	0.11	0.11	0.15	-0.01	0.01	0.05
	(0.38)	(0.36)	(0.20)	(0.96)	(0.95)	(0.70)
	[0.12]	[0.12]	[0.12]	[0.14]	[0.14]	[0.14]
	[-0.13,0.34]	[-0.13,0.35]	[-0.08,0.38]	[-0.29,0.28]	[-0.27,0.29]	[-0.22,0.32]
Household size	0.01	0.01	0.01	0.02	0.02	0.02
	(0.62)	(0.58)	(0.63)	(0.48)	(0.42)	(0.45)
	[0.02]	[0.02]	[0.02]	[0.03]	[0.03]	[0.03]
	[-0.04,0.06]	[-0.03,0.06]	[-0.03,0.06]	[-0.04,0.08]	[-0.03,0.08]	[-0.03,0.07]
House owned	-0.02	0.05	0.13	-0.47	-0.33	-0.23
	(0.93)	(0.85)	(0.56)	(0.08)	(0.23)	(0.38)
	[0.23]	[0.23]	[0.22]	[0.27]	[0.27]	[0.26]
	[-0.47,0.43]	[-0.42,0.51]	[-0.31,0.58]	[-1.00,0.06]	[-0.87,0.21]	[-0.76,0.29]

Rented house	0.01	0.04	0.10	-0.40	-0.35	-0.28
	(0.95)	(0.85)	(0.60)	(0.08)	(0.12)	(0.20)
	[0.19]	[0.19]	[0.19]	[0.23]	[0.23]	[0.22]
	[-0.36,0.39]	[-0.34,0.42]	[-0.27,0.47]	[-0.85,0.04]	[-0.80,0.10]	[-0.71,0.15]
Family owned house	0.11	0.14	0.18	-0.49	-0.42	-0.38
	(0.59)	(0.51)	(0.40)	(0.05)	(0.11)	(0.13)
	[0.21]	[0.22]	[0.21]	[0.25]	[0.25]	[0.25]
	[-0.31,0.54]	[-0.28,0.57]	[-0.24,0.59]	[-0.99,0.01]	[-0.92,0.09]	[-0.86,0.11]
Nb painted surfaces	0.03	0.03	0.03	-0.02	-0.03	-0.03
	(0.24)	(0.38)	(0.29)	(0.49)	(0.39)	(0.45)
	[0.03]	[0.03]	[0.03]	[0.03]	[0.04]	[0.03]
	[-0.02,0.09]	[-0.03,0.09]	[-0.03,0.09]	[-0.09,0.04]	[-0.10,0.04]	[-0.10,0.04]
Surveyor 2	0.15	0.15	0.12	-0.12	-0.12	-0.16
	(0.11)	(0.09)	(0.19)	(0.26)	(0.28)	(0.13)
	[0.09]	[0.09]	[0.09]	[0.11]	[0.11]	[0.10]
	[-0.03,0.32]	[-0.03,0.34]	[-0.06,0.29]	[-0.33,0.09]	[-0.33,0.10]	[-0.36,0.05]
Surveyor 3	0.51	0.53	0.55	-0.51	-0.49	-0.46
	(0.00)	(0.00)	(0.00)	(0.00)	(0.00)	(0.00)
	[0.12]	[0.12]	[0.12]	[0.14]	[0.14]	[0.14]
	[0.27,0.75]	[0.29,0.77]	[0.32,0.78]	[-0.79,-0.23]	[-0.77,-0.20]	[-0.74,-0.19]
Surveyor 4	0.27	0.27	0.18	0.18	0.19	0.08
	(0.00)	(0.00)	(0.05)	(0.10)	(0.08)	(0.44)
	[0.09]	[0.09]	[0.09]	[0.11]	[0.11]	[0.11]
	[0.09,0.45]	[0.09,0.45]	[0.00,0.36]	[-0.04,0.39]	[-0.02,0.40]	[-0.13,0.30]
Constant	-0.04	-0.08	-0.12	0.64	0.53	0.48
	(0.89)	(0.78)	(0.64)	(0.04)	(0.10)	(0.12)

	[0.27]	[0.27]	[0.26]	[0.31]	[0.32]	[0.31]
	[-0.56,0.49]	[-0.61,0.46]	[-0.64,0.39]	[0.02,1.27]	[-0.10,1.16]	[-0.13,1.09]
Out. mean (No Lead)	0.57	0.57	0.57	0.22	0.22	0.22
Observations	153	153	153	153	153	153

Note: This table reports the coefficients for the covariates in Table 2.

p-values are reported in parenthesis, standard errors and confidence intervals in square brackets.
