# Supplementary material for: Personalized lead exposure information and preventive behaviors in Ivory Coast: Insights from a pilot study
Source: PLoS One. 2025 Nov 14;20(11):e0336949. doi: 10.1371/journal.pone.0336949 (PMC12617878; doi:10.1371/journal.pone.0336949)
Supplement: S3 Table — Impact of tests’ results on awareness of lead exposure risks: panel specification. (PDF) [file pone.0336949.s004.pdf]

Robustness with panel specification.

Table 1: Impact of tests' results on awareness of lead exposure risks: panel specification

	Do you think that you are exposed to lead in your daily life?	
	Outcome: Yes	
	(1)	(2)
Lead detected	0.299***	
	(0.0645)	
Lead: Demonstration test only		0.304***
		(0.0710)
Lead: XRF only		0.285**
		(0.117)
Lead: XRF & Demonstration test		0.293***
		(0.104)
Individual FE	Yes	Yes
Wave dummies	Yes	Yes
Observations	519	519

* $p < 0.1$, ** $p < 0.05$, *** $p < 0.01$. Data from all three waves are included.
