# Supplementary material for: Personalized lead exposure information and preventive behaviors in Ivory Coast: Insights from a pilot study
Source: PLoS One. 2025 Nov 14;20(11):e0336949. doi: 10.1371/journal.pone.0336949 (PMC12617878; doi:10.1371/journal.pone.0336949)
Supplement: S4 Table — (PDF) [file pone.0336949.s005.pdf]

Table 1: Take-up of preventive measures: cleaning and renovating.

	Did you take any preventive measures?				
	Cleaning			Renovate	
	Clean surfaces	Clean doors & windows	Remove paint chips	Renovate	Careful during renovation
	(1)	(2)	(3)	(4)	(5)
Asset based wealth score	0.00	-0.01	0.01	0.00	0.00
	(0.94)	(0.59)	(0.44)	(0.94)	(0.73)
	[0.02]	[0.02]	[0.02]	[0.02]	[0.01]
	[-0.04,0.04]	[-0.06,0.04]	[-0.02,0.05]	[-0.03,0.03]	[-0.02,0.03]
No education (Head)	0.06	0.22	0.07	-0.08	-0.05
	(0.53)	(0.04)	(0.39)	(0.27)	(0.41)
	[0.09]	[0.11]	[0.08]	[0.07]	[0.06]
	[-0.12,0.23]	[0.01,0.43]	[-0.08,0.22]	[-0.22,0.06]	[-0.16,0.07]
Primary educ (Head)	0.08	0.11	0.10	0.00	-0.03
	(0.36)	(0.29)	(0.17)	(0.98)	(0.61)
	[0.09]	[0.10]	[0.07]	[0.07]	[0.06]
	[-0.09,0.25]	[-0.09,0.31]	[-0.04,0.24]	[-0.13,0.14]	[-0.14,0.08]
Sec. educ (Head)	-0.00	-0.07	-0.03	0.00	0.01
	(0.95)	(0.39)	(0.60)	(0.96)	(0.76)
	[0.07]	[0.08]	[0.06]	[0.06]	[0.05]
	[-0.14,0.14]	[-0.24,0.09]	[-0.15,0.09]	[-0.11,0.12]	[-0.08,0.11]
No educ (Woman)	-0.15	-0.08	0.06	-0.04	0.04
	(0.11)	(0.44)	(0.47)	(0.54)	(0.52)
	[0.09]	[0.11]	[0.08]	[0.07]	[0.06]
	[-0.33,0.03]	[-0.30,0.13]	[-0.10,0.21]	[-0.19,0.10]	[-0.08,0.16]
Primary educ (Woman)	-0.13	-0.14	-0.05	-0.06	-0.00
	(0.14)	(0.18)	(0.48)	(0.38)	(0.94)
	[0.09]	[0.10]	[0.07]	[0.07]	[0.06]
	[-0.30,0.05]	[-0.35,0.07]	[-0.20,0.09]	[-0.20,0.08]	[-0.12,0.11]
Sec. educ (Woman)	-0.20	-0.19	-0.01	-0.12	-0.02
	(0.02)	(0.05)	(0.83)	(0.08)	(0.68)
	[0.08]	[0.10]	[0.07]	[0.07]	[0.05]
	[-0.36,-0.04]	[-0.39,-0.00]	[-0.15,0.12]	[-0.25,0.01]	[-0.13,0.08]
35-30 years old	-0.01	0.04	0.01	0.09	0.00

	(0.89)	(0.65)	(0.79)	(0.10)	(0.93)
	[0.07]	[0.08]	[0.06]	[0.05]	[0.04]
	[-0.14,0.12]	[-0.12,0.19]	[-0.10,0.13]	[-0.02,0.19]	[-0.08,0.09]
30-35 years old	0.04	0.07	-0.07	0.04	-0.06
	(0.61)	(0.47)	(0.26)	(0.53)	(0.21)
	[0.08]	[0.09]	[0.06]	[0.06]	[0.05]
	[-0.11,0.19]	[-0.11,0.24]	[-0.20,0.06]	[-0.08,0.16]	[-0.16,0.04]
35-40 years old	0.12	0.17	0.16	0.15	0.03
	(0.20)	(0.11)	(0.04)	(0.04)	(0.65)
	[0.09]	[0.11]	[0.08]	[0.07]	[0.06]
	[-0.06,0.29]	[-0.04,0.38]	[0.00,0.31]	[0.01,0.29]	[-0.09,0.14]
40-45 years old	0.02	0.07	-0.01	-0.04	-0.07
	(0.84)	(0.64)	(0.94)	(0.72)	(0.40)
	[0.12]	[0.15]	[0.10]	[0.10]	[0.08]
	[-0.22,0.27]	[-0.22,0.36]	[-0.21,0.20]	[-0.23,0.16]	[-0.23,0.09]
Nb child. $\leq$ 5 yrs old	0.06	0.01	0.04	-0.02	0.06
	(0.23)	(0.83)	(0.33)	(0.61)	(0.09)
	[0.05]	[0.06]	[0.04]	[0.04]	[0.03]
	[-0.04,0.16]	[-0.10,0.13]	[-0.04,0.13]	[-0.10,0.06]	[-0.01,0.12]
Two months pregnant	-0.15	0.05	0.14	-0.15	-0.01
	(0.15)	(0.69)	(0.13)	(0.09)	(0.91)
	[0.11]	[0.12]	[0.09]	[0.08]	[0.07]
	[-0.36,0.06]	[-0.20,0.30]	[-0.04,0.31]	[-0.31,0.02]	[-0.14,0.13]
Three months pregnant	-0.04	0.04	0.22	-0.08	-0.05
	(0.67)	(0.74)	(0.00)	(0.29)	(0.39)
	[0.09]	[0.11]	[0.08]	[0.07]	[0.06]
	[-0.22,0.14]	[-0.18,0.25]	[0.07,0.37]	[-0.22,0.07]	[-0.17,0.07]
Four months pregnant	-0.15	-0.01	0.12	-0.12	-0.04
	(0.14)	(0.95)	(0.17)	(0.14)	(0.50)
	[0.10]	[0.12]	[0.08]	[0.08]	[0.06]
	[-0.34,0.05]	[-0.24,0.22]	[-0.05,0.28]	[-0.28,0.04]	[-0.17,0.08]
Five months pregnant	-0.01	0.08	0.18	-0.08	-0.02
	(0.93)	(0.49)	(0.04)	(0.33)	(0.81)
	[0.10]	[0.12]	[0.08]	[0.08]	[0.06]
	[-0.21,0.19]	[-0.15,0.31]	[0.01,0.34]	[-0.24,0.08]	[-0.14,0.11]
Household size	0.01	0.01	-0.01	0.03	-0.01

	(0.78)	(0.58)	(0.61)	(0.10)	(0.35)
	[0.02]	[0.02]	[0.02]	[0.02]	[0.01]
	[-0.03,0.04]	[-0.03,0.06]	[-0.04,0.02]	[-0.01,0.06]	[-0.04,0.01]
House owned	-0.07	-0.29	-0.14	-0.00	0.01
	(0.73)	(0.21)	(0.40)	(0.98)	(0.93)
	[0.19]	[0.23]	[0.16]	[0.15]	[0.12]
	[-0.45,0.32]	[-0.74,0.16]	[-0.46,0.19]	[-0.31,0.30]	[-0.24,0.26]
Rented house	0.10	-0.15	-0.10	0.05	0.02
	(0.53)	(0.42)	(0.44)	(0.68)	(0.86)
	[0.16]	[0.19]	[0.13]	[0.13]	[0.10]
	[-0.21,0.42]	[-0.52,0.22]	[-0.37,0.16]	[-0.20,0.30]	[-0.19,0.22]
Family owned house	0.12	-0.04	-0.07	-0.03	0.08
	(0.52)	(0.85)	(0.64)	(0.85)	(0.50)
	[0.18]	[0.21]	[0.15]	[0.14]	[0.12]
	[-0.24,0.47]	[-0.46,0.38]	[-0.37,0.23]	[-0.31,0.26]	[-0.15,0.31]
Nb painted surfaces	0.04	0.03	-0.00	0.04	-0.00
	(0.12)	(0.40)	(0.84)	(0.05)	(0.89)
	[0.03]	[0.03]	[0.02]	[0.02]	[0.02]
	[-0.01,0.09]	[-0.03,0.08]	[-0.05,0.04]	[-0.00,0.08]	[-0.04,0.03]
Surveyor 2	-0.13	-0.24	-0.10	0.03	0.00
	(0.10)	(0.01)	(0.13)	(0.64)	(0.92)
	[0.08]	[0.09]	[0.06]	[0.06]	[0.05]
	[-0.28,0.02]	[-0.41,-0.06]	[-0.22,0.03]	[-0.09,0.15]	[-0.09,0.10]
Surveyor 3	-0.16	0.45	-0.12	0.01	-0.01
	(0.11)	(0.00)	(0.16)	(0.86)	(0.84)
	[0.10]	[0.12]	[0.09]	[0.08]	[0.07]
	[-0.37,0.04]	[0.21,0.69]	[-0.29,0.05]	[-0.15,0.17]	[-0.14,0.12]
Surveyor 4	0.11	0.41	0.46	0.09	0.00
	(0.16)	(0.00)	(0.00)	(0.13)	(0.96)
	[0.08]	[0.09]	[0.06]	[0.06]	[0.05]
	[-0.04,0.25]	[0.24,0.59]	[0.34,0.59]	[-0.03,0.21]	[-0.09,0.10]
Constant	0.05	0.25	0.05	-0.07	0.05
	(0.83)	(0.35)	(0.81)	(0.71)	(0.72)
	[0.22]	[0.26]	[0.19]	[0.18]	[0.15]
	[-0.40,0.49]	[-0.27,0.77]	[-0.33,0.42]	[-0.42,0.29]	[-0.24,0.34]
Out. mean (No Lead)	0.07	0.35	0.11	0.04	0.00

Observations	153	153	153	153	153

Note: This table reports the coefficients for the covariates in Table 3.

p-values are reported in parenthesis, standard errors and confidence intervals in square brackets.
