# Supplementary material for: Personalized lead exposure information and preventive behaviors in Ivory Coast: Insights from a pilot study
Source: PLoS One. 2025 Nov 14;20(11):e0336949. doi: 10.1371/journal.pone.0336949 (PMC12617878; doi:10.1371/journal.pone.0336949)
Supplement: S5 Table — (PDF) [file pone.0336949.s006.pdf]

Table 1: Take-up of preventive measures: child related measures.

	Did you take any preventive measures?				
	Child related measures				
	No chip in mouth	Clean play	Change play	Wash hands	Cut Nails
	(1)	(2)	(3)	(4)	(5)
Asset based wealth score	-0.08	-0.01	-0.00	-0.10	-0.03
	(0.00)	(0.66)	(0.96)	(0.00)	(0.33)
	[0.03]	[0.03]	[0.03]	[0.03]	[0.03]
	[-0.13,-0.03]	[-0.06,0.04]	[-0.05,0.05]	[-0.16,-0.03]	[-0.08,0.03]
No education (Head)	-0.27	-0.06	-0.09	-0.27	-0.24
	(0.03)	(0.62)	(0.44)	(0.09)	(0.06)
	[0.12]	[0.12]	[0.12]	[0.15]	[0.12]
	[-0.52,-0.02]	[-0.31,0.19]	[-0.34,0.15]	[-0.58,0.04]	[-0.49,0.01]
Primary educ (Head)	-0.06	-0.01	-0.04	-0.29	-0.21
	(0.67)	(0.93)	(0.78)	(0.09)	(0.11)
	[0.13]	[0.13]	[0.13]	[0.16]	[0.13]
	[-0.32,0.21]	[-0.28,0.25]	[-0.30,0.22]	[-0.62,0.05]	[-0.48,0.05]
Sec. educ (Head)	-0.24	0.03	-0.16	-0.18	-0.17
	(0.02)	(0.78)	(0.11)	(0.16)	(0.08)
	[0.10]	[0.10]	[0.10]	[0.12]	[0.10]
	[-0.43,-0.04]	[-0.17,0.23]	[-0.35,0.04]	[-0.43,0.07]	[-0.37,0.02]
No educ (Woman)	0.11	-0.01	0.21	0.16	-0.24
	(0.44)	(0.97)	(0.13)	(0.37)	(0.09)
	[0.14]	[0.14]	[0.14]	[0.17]	[0.14]
	[-0.17,0.39]	[-0.28,0.27]	[-0.07,0.48]	[-0.19,0.51]	[-0.52,0.04]
Primary educ (Woman)	-0.15	0.01	0.08	-0.11	-0.26
	(0.22)	(0.93)	(0.49)	(0.47)	(0.03)
	[0.12]	[0.12]	[0.12]	[0.15]	[0.12]
	[-0.39,0.09]	[-0.23,0.25]	[-0.16,0.32]	[-0.41,0.19]	[-0.50,-0.02]
Sec. educ (Woman)	-0.16	-0.05	-0.13	-0.18	-0.32
	(0.17)	(0.68)	(0.25)	(0.23)	(0.01)
	[0.12]	[0.12]	[0.11]	[0.14]	[0.11]
	[-0.39,0.07]	[-0.28,0.19]	[-0.36,0.10]	[-0.47,0.11]	[-0.55,-0.09]
35-30 years old	-0.11	0.03	0.16	-0.02	0.04
	(0.27)	(0.73)	(0.12)	(0.85)	(0.69)

	[0.10]	[0.10]	[0.10]	[0.12]	[0.10]
	[-0.31,0.09]	[-0.17,0.23]	[-0.04,0.35]	[-0.27,0.23]	[-0.16,0.24]
30-35 years old	-0.14	0.09	-0.03	-0.27	-0.08
	(0.18)	(0.37)	(0.73)	(0.04)	(0.45)
	[0.10]	[0.10]	[0.10]	[0.13]	[0.10]
	[-0.34,0.07]	[-0.11,0.30]	[-0.23,0.17]	[-0.53,-0.02]	[-0.28,0.13]
35-40 years old	0.20	-0.09	0.01	-0.15	0.09
	(0.12)	(0.49)	(0.92)	(0.35)	(0.47)
	[0.13]	[0.13]	[0.13]	[0.16]	[0.13]
	[-0.05,0.46]	[-0.35,0.17]	[-0.24,0.27]	[-0.48,0.17]	[-0.16,0.35]
40-45 years old	-0.09	0.05	-0.06	-0.37	-0.16
	(0.51)	(0.71)	(0.65)	(0.04)	(0.25)
	[0.14]	[0.14]	[0.14]	[0.17]	[0.14]
	[-0.37,0.19]	[-0.23,0.33]	[-0.34,0.21]	[-0.72,-0.02]	[-0.44,0.12]
Nb child. $\leq$ 5 yrs old	-0.14	-0.12	-0.13	-0.25	0.10
	(0.11)	(0.17)	(0.12)	(0.03)	(0.25)
	[0.09]	[0.09]	[0.09]	[0.11]	[0.09]
	[-0.31,0.03]	[-0.30,0.05]	[-0.31,0.04]	[-0.47,-0.03]	[-0.07,0.27]
Two months pregnant	-0.07	-0.12	0.22	0.01	-0.06
	(0.59)	(0.34)	(0.08)	(0.97)	(0.64)
	[0.13]	[0.13]	[0.13]	[0.16]	[0.13]
	[-0.33,0.19]	[-0.38,0.14]	[-0.03,0.48]	[-0.31,0.33]	[-0.32,0.19]
Three months pregnant	-0.05	-0.13	0.20	0.21	0.03
	(0.60)	(0.22)	(0.05)	(0.10)	(0.77)
	[0.10]	[0.10]	[0.10]	[0.13]	[0.10]
	[-0.26,0.15]	[-0.33,0.08]	[0.00,0.40]	[-0.04,0.47]	[-0.17,0.23]
Four months pregnant	0.11	0.05	0.28	0.31	0.00
	(0.33)	(0.70)	(0.02)	(0.04)	(0.97)
	[0.12]	[0.12]	[0.12]	[0.15]	[0.12]
	[-0.12,0.35]	[-0.19,0.28]	[0.05,0.52]	[0.02,0.61]	[-0.23,0.24]
Five months pregnant	0.04	-0.10	0.25	0.09	-0.02
	(0.74)	(0.44)	(0.04)	(0.56)	(0.87)
	[0.12]	[0.12]	[0.12]	[0.15]	[0.12]
	[-0.21,0.29]	[-0.34,0.15]	[0.01,0.50]	[-0.22,0.40]	[-0.27,0.22]
Household size	0.01	0.01	0.04	0.01	0.01
	(0.77)	(0.68)	(0.10)	(0.60)	(0.68)

	[0.02]	[0.02]	[0.02]	[0.03]	[0.02]
	[-0.04,0.05]	[-0.03,0.05]	[-0.01,0.08]	[-0.04,0.07]	[-0.03,0.05]
House owned	0.06	0.09	-0.11	0.43	-0.12
	(0.82)	(0.74)	(0.67)	(0.21)	(0.65)
	[0.27]	[0.27]	[0.26]	[0.34]	[0.27]
	[-0.48,0.60]	[-0.45,0.63]	[-0.65,0.42]	[-0.25,1.11]	[-0.66,0.42]
Rented house	0.01	0.11	-0.23	0.47	-0.05
	(0.94)	(0.59)	(0.27)	(0.07)	(0.81)
	[0.21]	[0.21]	[0.20]	[0.26]	[0.20]
	[-0.40,0.43]	[-0.30,0.53]	[-0.63,0.18]	[-0.05,0.99]	[-0.46,0.36]
Family owned house	0.22	0.11	-0.17	0.70	-0.08
	(0.37)	(0.66)	(0.49)	(0.03)	(0.75)
	[0.24]	[0.24]	[0.24]	[0.30]	[0.24]
	[-0.27,0.71]	[-0.38,0.60]	[-0.65,0.31]	[0.09,1.31]	[-0.56,0.41]
Nb painted surfaces	-0.02	0.02	0.03	-0.08	0.03
	(0.69)	(0.58)	(0.50)	(0.10)	(0.50)
	[0.04]	[0.04]	[0.04]	[0.05]	[0.04]
	[-0.09,0.06]	[-0.06,0.10]	[-0.05,0.10]	[-0.18,0.02]	[-0.05,0.10]
Surveyor 2	-0.26	0.01	-0.15	-0.08	0.06
	(0.01)	(0.93)	(0.13)	(0.53)	(0.52)
	[0.10]	[0.10]	[0.10]	[0.13]	[0.10]
	[-0.46,-0.06]	[-0.19,0.21]	[-0.35,0.04]	[-0.33,0.17]	[-0.14,0.27]
Surveyor 3	-0.40	0.02	-0.21	-0.33	-0.01
	(0.00)	(0.87)	(0.11)	(0.04)	(0.94)
	[0.13]	[0.13]	[0.13]	[0.16]	[0.13]
	[-0.66,-0.14]	[-0.24,0.28]	[-0.46,0.04]	[-0.65,-0.01]	[-0.26,0.24]
Surveyor 4	0.25	0.19	0.41	0.30	0.13
	(0.02)	(0.06)	(0.00)	(0.02)	(0.20)
	[0.10]	[0.10]	[0.10]	[0.12]	[0.10]
	[0.05,0.45]	[-0.01,0.39]	[0.21,0.61]	[0.05,0.55]	[-0.07,0.33]
Constant	0.51	0.03	0.02	0.19	0.27
	(0.10)	(0.93)	(0.96)	(0.61)	(0.38)
	[0.30]	[0.30]	[0.30]	[0.38]	[0.30]
	[-0.10,1.12]	[-0.58,0.64]	[-0.58,0.61]	[-0.57,0.95]	[-0.34,0.87]
Out. mean (No Lead)	0.00	0.05	0.05	0.05	0.00
Observations	77	77	77	77	77

Note: This table reports the coefficients for the covariates in Table 4.

p-values are reported in parenthesis, standard errors and confidence intervals in square brackets.
