# Supplementary material for: Personalized lead exposure information and preventive behaviors in Ivory Coast: Insights from a pilot study
Source: PLoS One. 2025 Nov 14;20(11):e0336949. doi: 10.1371/journal.pone.0336949 (PMC12617878; doi:10.1371/journal.pone.0336949)
Supplement: Attachment — Submitted filename: Response_ref2.pdf [file pone.0336949.s008.pdf]

Response to Referee 2 for for "Lead risks and prevention: evidence from Ivory Coast." (PONE-D-25-27524), resubmitted to *PLOS ONE*

Thank you very much for your time and your report. In what follows, we list the main changes we have made before responding to your individual comments.

1 Main changes

1. We have added a table in the appendix (Table S1) and a paragraph at the end of the descriptive statistics section to explore and discuss the attrition we faced between Waves 1 and 3.

2. The conclusion now discusses the limitations highlighted by both referees, in particular the small sample size and the fact that the outcomes are self-reported.

3. While reworking and preparing the data files, we noticed a coding mistake that affected the variable *number of painted surfaces* for two of our respondents. We corrected this mistake, which led to slight changes in the coefficients reported in our regression tables. However, this correction does not affect our main findings and conclusion.

2 Responses to individual comments

1. *The study is however based on limited sample size and duration. The study is a "pilot intervention" with a small sample of 200 pregnant women, of whom only 153 were ultimately visited three times. This small scale and short duration (September 2023 to March 2024) may limit the generalization of the findings and their long-term impact.*

   Thank you for this comment. This study is indeed based on a pilot intervention involving a relatively small number of women over a short period of time. We nevertheless believe that it provides useful insights, which motivated us to prepare a paper and submit it for publication. While the sample size cannot be changed, we have added an attrition analysis to assess whether and to what extent attritors differ from non-attritors. In addition, the conclusion now highlights the importance of assessing whether behavioral changes are sustained over the long term, and of validating outcomes with objective measures such as children's blood lead levels, in order to establish whether personalized information leads to lasting health benefits.

2. *The study particularly relies on self-reported data. The behavioral changes are based on self-reported actions, such as hand washing and preventing children from eating paint chips. This method can be prone to bias, as participants might over-report positive behaviors.*

   You are right that self-reported behaviors are prone to bias. In the revised version, we have added a paragraph on limitations in the conclusion, where we mention the risk of social desirability bias. As noted above, the text now also emphasizes the importance of incorporating objective measures, which we plan to consider in the next phase of the project.

3. *There is a lack of long-term impact assessment. The paper explicitly states that more research is needed to understand if the observed behavioral changes persist over time and whether they have a tangible impact on children's blood lead levels. The study itself does not provide this crucial information.*

   The main goal of this study was to evaluate behavioral changes following personalized information on lead, but you are right that a reduction in children's blood lead levels is the ultimate outcome of interest. As part of this pilot, we obtained blood lead level (BLL) measurements for 28 children aged 9 months to 9 years, but we do not analyze this information here for two reasons. First, the sample is too small to allow for statistically robust analyses with sufficient power. Second, we do not expect to observe measurable effects on children over such a short time frame. In the next phase, our plan is to deploy the intervention on a much larger scale and to collect BLL data in order to evaluate the effect of personalized information on Pb exposure.

4. *The study shows no impact on key behaviors. While the intervention was successful in some areas, it had no impact on home-cleaning or renovation behaviors. The authors speculate this is due to the already high baseline frequency of cleaning and the high cost of renovations, but this still represents a limitation of the intervention's effectiveness.*

   The lack of effect on cleaning and renovation is indeed disappointing. In the follow-up study, we plan to provide incentives for renovation in order to reduce its actual cost. This should help clarify whether the absence of an effect in our study is related to the high cost of renovation

5. *The authors acknowledge that some women used the kit on their own and the estimated coefficient for this self-test could be biased because the choice to use it may be influenced by unobserved factors.*

   Yes, the coefficient on self-testing can only be interpreted as suggestive evidence. The design of the pilot study does not allow for a causal interpretation.

6. *Overall the authors has studied one of the primary public health concerns, however the small sample size has added a limitation to it and or its generalization of its findings.*

   Thank you for this comment. We now clearly state in the conclusion, along with the other limitations, that the small sample size limits the generalization of the findings.
