# Supplementary material for: Personalized lead exposure information and preventive behaviors in Ivory Coast: Insights from a pilot study
Source: PLoS One. 2025 Nov 14;20(11):e0336949. doi: 10.1371/journal.pone.0336949 (PMC12617878; doi:10.1371/journal.pone.0336949)
Supplement: Attachment — Submitted filename: review comments_lead.docx [file pone.0336949.s007.docx]

**Summary:**

This manuscript presents a pilot intervention in Abidjan, Ivory Coast, evaluating whether providing personalized information about lead (Pb) presence in household paint can improve awareness and promote preventive behaviors among pregnant women. The study involved testing painted surfaces in 200 homes using a low-cost Pb detection kit and follow-up XRF analysis. Results showed that women who received personalized information were significantly more likely to recognize lead risks and report behavior changes (e.g., preventing children from ingesting paint chips, increased handwashing). However, no measurable impact was observed on cleaning or renovation practices. The study concludes that personalized, low-cost detection tools can effectively raise awareness and potentially reduce Pb exposure in vulnerable populations. I have listed some concerns and suggestions below that should be clarified to improve the manuscript’s current state.

**Detailed comments:**

1. The title is concise and relevant, but it could better capture the novelty of the intervention explicitly. Consider making it more specific, such as saying that “Personalized Lead Exposure Information Improves Preventive Behaviors in Abidjan: Evidence from a Pilot Study.” Consider exactly or close.
2. The abstract effectively summarizes the study, but it should include the actual number of participants who completed all visits (153), and clarify that behavioral outcomes were self-reported because it may probably limit objectivity.
3. In the introduction, the global burden of lead exposure is well established. However, the reference to the CDC’s “old reference level” of 5 µg/dL should be clarified by explaining that the CDC now uses a blood lead reference value of 3.5 µg/dL, and whether this impacts comparability in recent time.
4. The sentence in line 18, “our own 2023 testing,” is too informal for scientific writing. Maybe you can better say that... “A 2023 assessment conducted by the authors.”
5. The paragraph discussing the cost of XRF and the benefits of the new kit (lines 33 to 39) would benefit from providing approximate costs or operational advantages and more to better contextualize their practicality.
6. The methods section is well organized, but attrition from 200 to 153 participants should be formally addressed. An attrition analysis should be included to examine potential bias between those who completed all visits and those who did not.
7. The explanation of the consent process should be revised for clarity. “Selecting OK or signing on the device” is too casual. It should state that written and digital consent was obtained through signed forms or the secure electronic signatures.
8. There is need for more technical details about the detection kit should be adequately provided, including its major details like the sensitivity, specificity, manufacturer, and performance validation to support reproducibility. This is the most important out of all and should be adequately attended to.
9. In lines 74–77, the call for future research should be expanded. Specifically mention the need for studies that assess long-term behavioral change and validate outcomes with blood lead measurements in children.
10. A formal limitations paragraph is missing and should be included. There should place to acknowledge the reliance on self-reported data, the absence of biological markers (e.g., blood lead levels), potential Hawthorne effects, and attrition bias.
11. Several sentences begin with conjunctions such as “However” or “But,” which can be smoothed out for more professional tone. For example,.. “However, we find...” could become “We found no evidence that….” Just to be very direct and whatnot
12. Reference [27] (Benjamini et al.) is correctly cited to justify controlling false discovery rates, but a short sentence in the methods should be added to explain why this method was selected over others, such as Bonferroni or Holm, the stepwise.
13. The authors’ mention of ChatGPT for language editing is transparent and ethical, but it may be preferable to rephrase as: “Language editing was assisted by ChatGPT (OpenAI). The authors reviewed and approved all content to ensure scientific accuracy and accept full responsibility for the manuscript.”
14. Self-use of the kit is included in robustness checks, but because usage was not randomized, the results from self-use should be described more cautiously. Asuthors should reinforce the limitation more strongly in the results and discussion.
15. The sample size, particularly for behavior outcomes involving only households with children under 5, is relatively small (n = 77). I think this limitation is best to be clearly stated in the discussion as it affects statistical power and generalizability.
16. The policy implications are sound, but they could be made more specific. The authors might propose integration of low-cost Pb detection kits into routine maternal and child health outreach or suggest partnerships with municipal housing authorities.
